# AI-mediated intergenerational skill transfer: enhancing ESG literacy and sustainable consumption among aging populations

**DOI:** 10.3389/frai.2026.1857373

**Published:** 2026-08-19

**Authors:** Anuja Paul, Astle, Sujata Roy

**Affiliations:** 1 Manipal Law School, Manipal Academy of Higher Education, Manipal, India; 2 Gitam School of Law, Gandhi Institute of Technology and Management, Visakhapatnam, India; 3 The West Bengal National University of Juridical Sciences, Kolkata, India

**Keywords:** ageing, artificial intelligence, ESG, SDG 12, skill transfer

## Abstract

As nations face two significant megatrends—demographic aging and digital transformation—there is a need to align technological innovation with overarching sustainability goals. This study examines the application of Artificial Intelligence (AI) to improve intergenerational skill transfer, thereby enabling aging populations to substantially contribute to the enhancement of Environmental, Social, and Governance (ESG) literacy and Sustainable Development Goal 12 (Responsible Consumption and Production). This examines the potential collaboration between AI-driven lifelong learning platforms and the engagement of elderly individuals as mentors in sustainability education. The study is guided by three primary inquiries: How can AI-assisted learning environments enhance the capacity of older individuals to transmit ESG-related skills? What impact do AI-personalized, senior-led sustainability programs have on the behaviors and attitudes of younger learners? What ethical and design issues are essential to ensuring that AI technologies equitably support older educators in sustainability transitions? This study uses a stepwise mixed-methods approach, beginning with a thorough assessment of AI applications in adult and intergenerational ESG education. Following this, quantitative data will be collected through interviews with elder participants using AI-assisted sustainability platforms, in conjunction with the qualitative analysis of pre- and post-intervention data to assess environmental attitudes among youth learners. The objectives of this study are to explore how AI-assisted learning environments enhance the capacity of elderly individuals to transmit ESG-related skills and to identify what ethical and design challenges are essential to ensure that AI technologies equitably support elderly individuals in transitioning to sustainability practices. This qualitative study addresses a research gap in AI for Good applications for inclusive environmental governance and digital aging. This study contributes to the expanding domains of AI for good, inclusive environmental governance, and digital aging by presenting a novel framework that integrates human knowledge with machine intelligence to foster sustainable futures.

## 1 Introduction

“The integration of ESG ethical principles into SDG 12 is not merely about reducing waste or promoting sustainability, but more about empowering the elder generation as trailblazer for intergenerational responsibility- the consumption, choices and lived experience adds up in the community to contribute in effective, inclusive and circular economy with governed ethics.”

*Journal of Sustainable ageing and policy, 2023 (editorial note).*

The world faces intersecting megatrends of demographic aging and digital transformation, necessitating alignment of AI innovations with long-term sustainability goals (Wang et al., 2024; Li et al., 2021; Liao et al., 2023). This study explores how AI can facilitate intergenerational skill transfer, positioning aging populations as mentors in Environmental, Social, and Governance (ESG) literacy and SDG 12 (responsible consumption and production) Globally, the social and environmental landscape is changing as the 21st century progresses due to two revolutionary megatrends: demographic aging and digital transformation (United Nations, 2015). Although technological progress, especially artificial intelligence (AI), has the potential to increase sustainability and efficiency, little is known about how it aligns with social equality and intergenerational justice. At the same time, the elderly population, which is frequently left out of the digital economy, possesses substantial implicit knowledge essential to facilitating the fair transition to sustainability.

The article adopts a perspective on assumptions about age-related “capabilities” in the later stages of life. Demographic aging underpins the concept of AI-driven technology envisaged for an intergenerational learning process that supports an ethical economy through ESG awareness and education. Although overall wellbeing requires contributions from all sectors of society, irrespective of gender, age, and other determinants, the study generally explores through the lens of ageism, which shall also be inquisitive determinants and limitations of the paper. The growing need for improvement in the environmental, social, and governance model (ESG) also aligns with SDG 12 (responsible consumption and production); in that pursuit, skill transfer methods and creative educational strategies should be paramount (Lorek and Wahlen, 2012).

The central theme is technology, which could be imperative to eradicate ageism and promote inclusivity through AI, addressing a righteous research gap in prioritizing technological solutions while simultaneously addressing social evil that affect the wellbeing of elderly adults (Battisti, 2023). Specifically, this approach could enhance productivity by positioning older adults as mentors and skills-transfer agents, thereby ensuring social determinants of health, productivity, and an uncompromised effective economy. The effectiveness could be enhanced by imbibing AI modeled-mediated technology, potentially exacerbating existing inequality and advocating for a sustainable and equitable future for all. This article presents systematic evidence to identify, facilitate, and examine the range of digital skills in serving the United Nations Goals (UNG). After screening, the study primarily focused on ESG education through AI among youths mentored by skillful elders (Helsper et al., 2021). The analysis of skewness and kurtosis has been substantiated through cognitive semi-structured interviews and literature surveys. The identified dimensions have been based on different contexts, encompassing subskills and digital knowledge that could result in the well-being of digital society.

The research questions are as follows: 1. How can AI-assisted learning environments enhance the capacity of older individuals to transmit ESG-related skills? 2. What impact do AI-personalized, senior-led sustainability programs have on the behaviors and attitudes of young learners? 3. What ethical and design issues are essential to ensure that AI technologies equitably support older educators in sustainability transitions?

The objectives of this study are to explore how AI-assisted learning environments enhance the capacity of elderly individuals to transmit ESG-related skills, and to identify what ethical and design challenges are essential to ensure that AI technologies equitably support elderly individuals in transitioning to sustainable practices. This qualitative study addresses a research gap in AI for Good applications for inclusive environmental governance and digital aging. The upcoming sections of this article are structured as follows: Section 2, *Survey of Literature and Rationale to Integrate SDG 12*, presents a survey of literature, which delineates more on the theoretical framework and sets the precision in the research gap and policy suggestions. Section 3, *Subject and Method*, elucidates the data collection through semi-structured interviews, including sampling and thematic analysis, of elderly individuals in the context of intergenerational justice. Section 4, *Confluence of ESG through the AI Hub: Ensuring Inclusivity of Digital Natives and Digital Immigrants toward SDG 12*, discusses advancements in inclusivity through AI. Section 5, *Results and Discussion*, presents key findings, discussions, and analysis characterizing SDG footprints (12) and ESG awareness. Section 6, *Conclusion and Policy Implication*, concludes with details on implications and further scope of the study.

## 2 Survey of literature and rationale to integrate SDG 12

### 2.1 Conceptual framework on ESG and SDG 12

The integration of social, environmental, and governance practices into policy is of the utmost importance for transparency and accountability. Transparency and accountability can only be ethical when they align with the United Nations’s SDGs. A critical issue to consider is that numerous efforts and approaches have been developed to align SDGs and ESGs; however, these efforts are often overlooked by corporates, and their significance is not sufficiently recognized by academicians (Hartley et al., 2023). Studies have propounded the impact of ESG on SDGs within corporate settings (Chien et al., 2023), whereas some studies have elaborated the negligible connection between ESGs and SDGs through quantitative analyses (Kocmanová and Dočekalová, 2012). Yoon et al. (2018) investigated South Korea’s ESG vision and identified significant links with SDGs by enhancing stakeholder perceptions. Emerging economies, such as Brazil, India, and China, have shown great resilience in environmental factors associated with SDGs, although their frameworks for Social and Governance remain limited (Soni, 2023). ESG disclosure impacts the country’s ranking in SDG indicators (Plastun et al., 2020). The metrics of stakeholders’ economy are linked to the governance dimensions of ESG to SDG 12 (responsible consumption and production) (World Economic Forum, 2020). These efforts should align with ESG disclosure practices to facilitate the mooting on SDGs (Eccles and Viviers, 2011).

### 2.2 Digital native and digital immigrants through responsive AI model

There are aligned themes between elderly populations and SDG goals, and stakeholder engagement from the perspective of social dimensions is paramount (Tamimi and Sebastianelli, 2017). The Global Reporting Initiative (2022) suggested that ESG responses should be appropriate, as they are directly proportional to enterprise growth and are largely influenced by social dynamics. Markopoulos and Markopoulos (2023) proposed a learning model in which the native generation of ubiquitous technology is multimodal and interactive, whereas immigrants continue to face challenges in adopting the technology and face barriers in expression. Critical analysis has highlighted the potential of digital methods as learning tools for the disruption of intergenerational inequalities or reinforcing them. Digital natives navigate emerging technology with ease, whereas digital immigrants require assistance. The study also envisages the need for AI-based models to ensure usability while promoting social and digital equity (Peterson, 2025).

#### 2.2.1 AI adoption in education

Nassanbekova et al. (2026) used an integrated TAM-TRI approach to understand the student adoption of generative AI (GenAI) chatbots in education, highlighting behavioral, cognitive, and ethical factors relevant to intergenerational AI-mediated learning.

#### 2.2.2 AI for sustainability

Alsharaideh et al. (2026) extended dynamic capabilities theory to examine AI-enabled innovations for sustainable development.

#### 2.2.3 On digital gaps

Aumeboonsuke and Caplanova (2026) analyzed determinants of the digital divide between Central Europe and Southeast Asia, underscoring the barriers faced by “digital immigrants.”

Shojaei and Barbosa (2026) closely examined how Vietnamese e-commerce shoppers make decisions about last-mile deliveries. Using the stimulus-organism-response framework, they found that, when consumers perceive an option as more sustainable, their overall attitude toward it tends to improve. Cost, on the other hand, has the opposite effect.

#### 2.2.4 Consumer sustainability and the role of awareness and literacy

Khuadthong et al. (2025) explored the factors influencing sustainable tourism behaviors, such as choosing eco-friendly accommodations, low-emission transportation, and responsible destination practices. This study complements consumer sustainability studies by extending the focus to tourism behaviors. It reinforces the role of awareness and literacy as key drivers of sustainable choices alongside cost and convenience considerations.

Islam et al. (2026) introduced a comprehensive simulated AI-Driven IoT dataset specifically designed for remote health monitoring and fall detection among elderly individuals. The dataset integrates data from multiple IoT sensors (e.g., wearable accelerometers, gyroscopes, heart rate monitors, and environmental sensors) to capture physiological signals, movement patterns, and daily activities.

Chaouali (2025) investigated the resistance of elderly customers toward AI-powered chatbots in the banking sector. The study explores psychological, technological, and social barriers that prevent older adults from adopting conversational AI for banking services, such as customer support, transactions, and financial advice. This research complements studies on AI adoption in education (Nassanbekova et al., 2026) and consumer behavior (Shojaei and Barbosa 2026).

## 3 Subject and method

The study used a qualitative descriptive approach using purposive sampling and semi-structured interviews. Participants (*N* = 14, aged 60–75) were selected based on the following criteria: (a) basic digital literacy, (b) high cognitive function and motivation for sustainability-related topics, and (c) professional backgrounds (e.g., professors, retired defense officers, healthcare specialists, senior association members). Selection occurred through university networks and senior citizen groups based in the metropolitan cities of India.

### 3.1 Study population

Elderly individuals aged 60–75 were recruited for the study. The intention was not to consider them as participants but as active digital inmates for transmitting digital module education focusing on ESG. The age criterion is standard and in concordance with World Health Organization reports. These individuals demonstrated high cognitive abilities to qualify as “mentors,” possessed basic digital literacy, and were moderately to highly proficient in the primary language, typically English. They expressed their motivation toward sustainable development goals and aligned areas. Participants included university professors, healthcare specialists, senior citizen associations, and individuals with high social acceptance.

### 3.2 Data analysis

Thematic analysis (Braun and Clarke, 2006) was conducted using NVivo (Lumivero, 2015). Transcripts were coded iteratively to identify themes such as willingness, barriers, knowledge transfer, and ESG commitment. Trustworthiness was ensured through member checking (where feasible), triangulation with existing literature, and reflexivity. Limitations of the small sample size and qualitative nature are acknowledged regarding generalizability.

Sample size.

*N* (number of individuals) = 14.

Considering the requirements of digital literacy and willingness to engage on the topic of sustainability, the numbers are apt to assist the research in depth and focus.

Informed consent was obtained before the interview. The interview session lasted for 15–20 min and was transcribed in the same way for analysis. The semi-structured question and consent form are attached to this study. The interview was conducted in English, and most participants were comfortable communicating in this language.

## 4 Confluence of ESG through AI hub: ensuring inclusivity of digital natives and digital immigrants toward SDG 12

**Figure 1:**
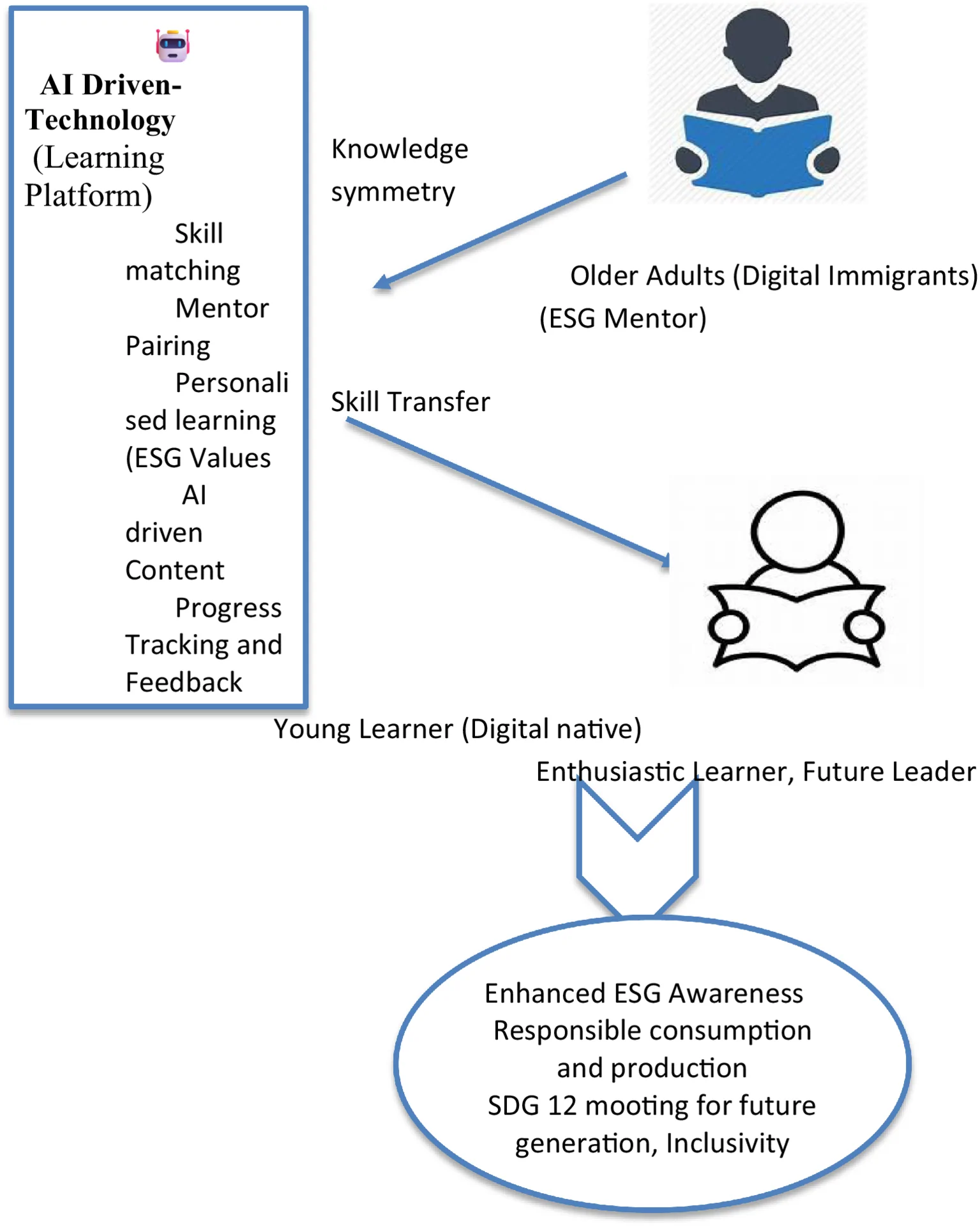
AI-mediated framework for intergenerational learning (Source – visualized by authors for AI-mediated training, only for the purpose of academic literature).

As conceptualized in (Figure 1), AI-mediated intergenerational ESG learning emphasizes the central subject of the study, which is leveraging artificial intelligence (AI) to facilitate intergenerational ESG (Environmental, Social, and Governance) mooting on Sustainable Development Goals 12 (SDG - Responsible consumption and Production). The model connects “digital immigrants” to digital natives, linking the older generation to the younger one to facilitate their learning in ESG values. The older generation contributes impeccable experience and mentorship skills with real-life anecdotes, whereas younger individuals contribute curiosity, courage, and leadership in next-generation digital fluency (De Lucia et al., 2024). Artificial intelligence mediates the flow of skills and knowledge. The AI-mediated hub supports the following functions:

- Leveraging technology to facilitate the flow of knowledge and learning
- AI-driven content curation and mentor pairing
- Personalized and effective ESG values
- Promoting ESG knowledge and awareness through a continuous feedback mechanism.

AI is evolving and, when aligned with ethics and inclusivity, can bridge the gap between demographic and digital divides (Vinuesa et al., 2020). The conceptualized AI-mediated technology could strengthen ESG education and intergenerational collaboration but should be guided by ethical principles and rules of equity (UNESCO, 2020). Moreover, the proposed model positions AI as a guiding tool for intergenerational skills and knowledge transfer.

The integration of SDG 12 ensures profit, sustainable governance, and the minimization of environmental footprints with the utmost resource utilization. The older population is faced with two-fold challenges on the economic front and resource allocation: uneven demographic dividends and a lack of digital awareness, resulting in significantly uneven wealth distribution. Thus, a well-established model could assist the different demographic sectors and create opportunities to address the issues related to the elderly population (Park and Jang, 2021). The traditional ESG model, focusing on environment and governance, with a major focus on social factors such as healthcare, could be the targeted intervention to enhance the quality of life and community engagement. Addressing the challenges of the aging population through ESG by mooting for SDG 12 could be advocated through a significant framework that has components in the environment, comprising waste management, GHG emission control (Peterson, 2025), and carbon footprint. In a corporation, aligning the governance model with ethical principles and corporate strategies in bilateral and multilateral investment treaties would ensure the effectiveness of social integration and inclusivity of the elderly population. This would enhance community engagement, providing ancillary services (Gopal and Pitts, 2025) to the aging population, such as healthcare benefits, infrastructure benefits, and institutional collaborations.

## 5 Results and discussions

This section analyzed and presented the descriptive analysis using qualitative methods to decipher the responses of participants regarding AI modulation and ESG. The study involved elderly individuals who were recruited to transmit digital education modules focusing on ESG and SDGs.

Participants expressed curiosity and willingness toward the AI-assisted education model and were motivated by their mentor roles. Although most participants initially hesitated regarding the operability of digital gadgets, Respondent 3 (n 3) mentioned being skeptical about using computers for a long time due to health issues. One derivation was that individual hesitancy is not solely attributable to non-operability or digital literacy but also to health concerns.

During discussions over sustainable development goals, a majority of the participants showed strong commitment to learning in support for different SDG goals. Healthcare providers and academicians vouched for SDGs 3, 6, and 12, which are good health, clean water, and responsible consumption, respectively.

Respondents 1 (n1) and n5 emphasized the need to transmit traditional knowledge to digital inmates as necessary for generation. 80% of the respondents (11 out of 14) agreed on the importance of technology to enhance education and advocated it as the sole and best method for disseminating information.

### 5.1 Analysis

The finding suggested the following:

- Commitment of elderly individuals to substantiate existing information with traditional and skillful inference.
- ESG commitment to intergenerational justice and knowledge transfer.
- Reluctance was shown on health grounds for consistency using the digital method (see Table 1).
  The table is a comparative, visual analysis of environmental, social, and governance knowledge; its relevance to SDG goals (specifically SDG 12); and its impact on older adults. According to the collected data, older adults are most likely to be affected by health hazards because of degrading environments, representing the environmental dimension (E). Inclusion programs in a corporate or entity promote experience and accountability in governance (Park and Jang, 2021); that is the governance dimension (G), and the social community order and initiatives ensure social sustainability and build a support system for lower-income seniors (De Lucia et al., 2024).

**Table 1:** Demonstration of ESG knowledge and its impact/relevance on various aspects.

ESG component pillars	Description	Relevance to SDG’s	Impact on elderly people
Environmental resource depletion	Environmental management and consumption of natural resources	SDG 12 responsible consumption and production and SDG 3 good health and wellbeing	Good public health and sensitization programs, wellbeing of elderly people’s health by reducing environmental stressors.
ECO products, GHG emission	Innovation for efficient methods of production in alignment with SDG 12, reducing emissions to ensure climate targets	SDG 3 Good health SDG 12 Production SDG 13 Climate Action	Energy-efficient products for senior citizens and a healthy, non-compromised environment.
Elder inclusivity, social community relations	In a workspace, it includes diversity and experience, which in turn ensures overall development Social community relations help to upscale the quantum of social responsibility	SDG 10 reduced inequality SDG 8 decent work and economic growth	Addresses and eradicates social isolation Ensures diversity and wellbeing in workspaces for older adults Upscale the youth and uplift the elderly community. Builds a support system for lower-income seniors.
Shareholder rights and ethical conduct	Transparent Corporate Governance ensuring safeguards for the shareholders’ interest, Assisting in due diligence	SDG 8 – decent work and economic growth SDG 5 – gender equality SDG 16 - peace justice and strong institute	Secures veracity among people for investment purposes, standardizes ethical conduct in a corporate governance model (Wang et al., 2024)

## 6 Conclusion and policy implication

The study examines the impact of ESG awareness on the disclosure of SDG 12, specifically across a broader spectrum, along with the AI-modulated role of the older generation passing skills and education to younger ones. The determinants showed that strong ESG awareness impacts the social setup and promotes inclusivity in the workplace for older adults. It also influences the younger generations (digital natives) to be the trailblazers in the entity (de Jesus and Nascimento, 2021). The key message drawn from the research is that older adults are not reluctant to use artificial intelligence; however, the health component is a paramount, energy-efficient product (Eldowma et al., 2023), which aligns with SDG 12 (Consumption). In our study, we addressed concerns related to positioning older adults as mentors within an AI-enabled module system for ESG awareness, aligned with SDG-focused education. The results demonstrated enriched intergenerational justice and promoted digital learning among younger generations. A majority of respondents mooted sustainable development goals; however, health-related concerns and varying digital comfort are key factors for long commitments. The proposed visualized system (Figure 1) is intended solely to assist and transfer skills and learning from the older generation to the younger ones.

### 6.1 Limitations and avenues for future research

Although the study offers novel and practical insights into the role of older adults as mentors in AI-mediated ESG awareness, a few limitations should be acknowledged. First, as the sample size was relatively small (*n* = 14) and geographically narrow, the findings could be general and niche; although the sample was considered based on reliance on technology (digital inmates), the findings may predominantly reflect more circumstantial value in the broader spectrum. Second, the study primarily relied on qualitative data, through semi-structured interviews, which may not reflect the behavioral diaspora and may be subject to desirability bias. Third, while the study has visualized the long-term impact, they remain to be deciphered. Health engagement and consistency also act as keys, as medical experts shall substantiate well to know the long-term commitment to effective learning.

Further research avenues for the readers could include expanding the demographic and gender diversity (SDG 6) and exploring its grandeur to frame a policy involving academicians, entities, and government intervention for cognitive and adaptive AI. Comparative and cultural values could be eccentric subjects that promote intergenerational justice and sustainable values. A more comprehensive body of literature could be developed using a longitudinal method as a research design, which should be appropriate for advancing the available literature and exploring the arena of cultural values and traditional knowledge, thereby promoting ecocentrism and integrating young and elder generations into a metro-sustainable education strategy.

## Data Availability

The raw data supporting the conclusions of this article will be made available by the authors, without undue reservation.
